# Study on Chemical Profile and Neuroprotective Activity of *Myrica rubra* Leaf Extract

**DOI:** 10.3390/molecules22071226

**Published:** 2017-07-24

**Authors:** Pinghong Chen, Xianzong Lin, Ching-Hsu Yang, Xu Tang, Yu-Wei Chang, Weibing Zheng, Lianzhong Luo, Changan Xu, Yung-Husan Chen

**Affiliations:** 1 Department of Pharmacy, Xiamen Medical College, Xiamen 361023, China; chenpinghong@tio.org.cn (P.C.); lintcm2014@163.com (X.L.); lzluo@xmu.edu.cn (L.L.); 2 Engineering Research Center of Marine Biological Resource Comprehensive Utilization, Third Institute of Oceanography, State Oceanic Administration, Xiamen 361005, China; tangxu@tio.org.cn; 3 Fineboon Dairy Nutrition Institute, Shaanxi Dairy Co., Ltd., Xianyang 712000, China; lukeyang2004@gmail.com; 4 Department of Food Science, National Taiwan Ocean University, Keelung 202, Taiwan; bweichang@email.ntou.edu.tw; 5 Key Laboratory of Marine Biogenetic Resources, Third Institute of Oceanography, State Oceanic Administration, Xiamen 361005, China; wbzhen@tio.org.cn; 6 Xiamen Key Laboratory of Marine Medicinal Natural Products and Cell Engineering, Xiamen Medical College, Xiamen 361008, China; 7 Key Laboratory for Dao-Di Herbs Biotechnology of Fujian Province, Xiamen Medical College, Xiamen 361023, China

**Keywords:** UPLC-PDA-HRMS, *Myrica rubra*, neuroprotective activity

## Abstract

The chemical profile of *Myrica rubra* (a native species in China) leaf extract was investigated by UPLC-PDA-HRMS, and the neuroprotective activity of two characteristic constituents, myricanol and myricetrin, was evaluated with N2a cells using H_2_O_2_-inducedoxidative challenge through a series of methods, e.g., MTT assay, ROS assay and [Ca^2+^]i assay. Among the 188 constituents detected in the extract of *Myrica rubra* leaf, 116 were identified definitely or tentatively by the comprehensive utilization of precise molecular weight and abundant multistage fragmentation information obtained by quadrupole orbitrap mass spectrometry. In addition, 14 potential new compounds were reported for the first time. This work established an example for the research of microconstituents in a complex analyte and revealed that suppression of H_2_O_2_-induced cytotoxicity in N2a cells was achieved by the pretreatment with myricanol. The evidence suggested myricanol may potentially serve as a remedy for prevention and therapy of neurodegenerative diseases induced by oxidative stress.

## 1. Introduction

*Myrica rubra* is a subtropical perennial plant belonging to the *Myricaceae* genus [1], and widely distributed in the east of Asia, e.g., China, Japan and Korea. For a long period of time, it had been used as traditional medicine for the treatment of skin diseases and diarrhea [2]. Moreover, it could be taken orally for traumatic injury, bone fracture, diarrhea, stomach and duodenal ulcer in ethno-medicine [3]. Various phytochemicals isolated from the leaves of *Myrica rubra* have been extensively investigated and generally reported to show numerous bioactivities, such as melanin synthesis-inhibitory [4], antitumor [5], and anti-influenzavirus activity [6]. However, there had been little information concerning the possible neuroprotective activity of *Myrica rubra*.

A comprehensive chemical profile of *Myrica rubra* has not been reported. Instead, there are some reports focused on its low polarity constituents—sesquiterpenes—and their pharmacological activities [7,8]. Considerable research has been directed towards the identification and characterization of anthocyanins and flavonols [9,10], aiming at the discovery of constituents with radical scavenging activity, especially for the major ones which are easy to isolate and purify. Nevertheless, its microconstituents which may play an important role in the biological system still need to be discovered. Consequently, a sensitive tool should be applied to achieve this goal which calls for little amounts of analytes.

UPLC-PDA-HRMS is an emerging technology widely used to analyze complex samples, owing to its high resolution and sensitivity. It not only provides exact mass values (<6 ppm) with possible molecular formulae but also abundant MS^2^ fragmentation information, integrated with UV spectra, allowing one to unravel the structural identification based on the Chemical Abstracts database (https://scifinder.cas.org/). In this work, UPLC-PDA-HRMS was used to analyze the extract of *Myrica rubra* leaf. The main and typical constituents in *Myrica rubra* leaf were flavonoids and cyclic diarylheptanoids. They had both been reported to exhibit neuroprotective activity [11,12,13,14,15,16], even though *Myrica rubra* had not.

Neurodegenerative disorders are considered to be associated with elevated oxidative stress levels [17], which play a vital role in the regulation of redox reactions and generation of reactive oxygen species (ROS) [18]. H_2_O_2_ is a well-known neurotoxin that triggers oxidative stress and apoptosis in neuronal cells by producing ROS. H_2_O_2_-induced cytotoxicity is thus a common method applied for the measurement of potential neuroprotective antioxidants [19,20,21]. H_2_O_2_ readily penetrates into cells and generates highly reactive hydroxyl radicals (a kind of ROS), thus inducing oxidative damage by attack on cellular components [22]. Previous reports have indicated that high-concentrations of H_2_O_2_ may cause the transformation of intracellular calcium [23] which could trigger the elevation of Ca^2+^, and excessive mitochondrial Ca^2+^ accumulation may give rise to superoxide anion production [24]. Antioxidants protect cells from massive oxidative stress by neutralizing ROS [25]. In this work, two representative constituents identified and isolated from *Myrica rubra* leaf extract, myricetrin (a flavonoid) and myricanol (a cyclic diarylheptanoid), were chosen to evaluate their neuroprotective effects against H_2_O_2_-induced cytotoxicity in N2a mouse neuroblastoma cells (N2a cells) via monitoring cell viability by a MTT (3-(4,5-dimethylthiazol-2-yl)-2,5-diphenyltetrazolium bromide) assay [26], intracellular ROS levels, and intracellular calcium ion ([Ca^2+^]i) influx [27].

## 2. Results and Discussion

**Figure 1:**
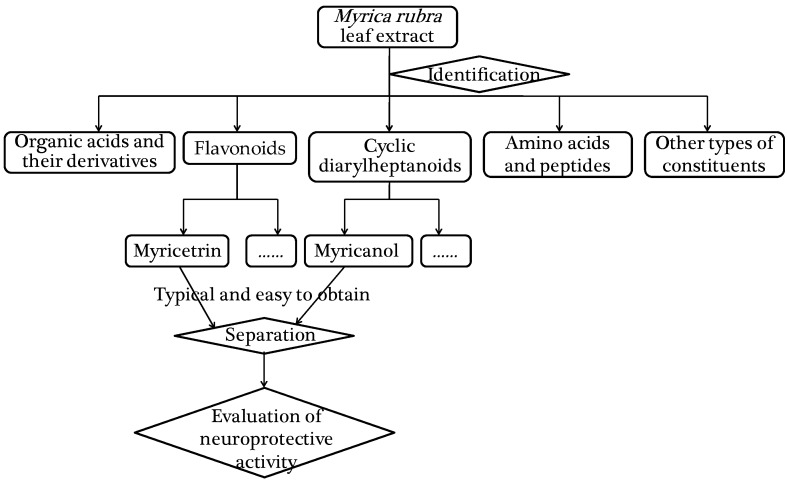
The schematic diagram of the proposed approach.

The widespread use of Myrica rubra in folk medicine has motivated intensive exploration of its pharmacological activities. Prior to that, the identification of constituents is of great importance and the chemical profile should be studied. Then the typical constituents were chosen to evaluate their neuroprotective activity. An approach based on UPLC-PDA-HRMS analysis and neuroprotective activity evaluation was proposed. The schematic diagram is illustrated in Figure 1.

### 2.1. Identification of Constituents in the Whole Extract of Myrica rubra

**Figure 2:**
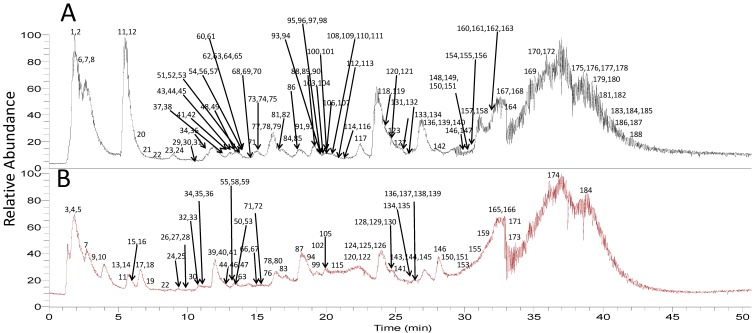
Total ion chromatograms (TICs) of the extract of *Myrica rubra* by UPLC-PDA-HRMS. (**A**) Negative ion mode; (**B**) positive ion mode.

**Figure 3:**
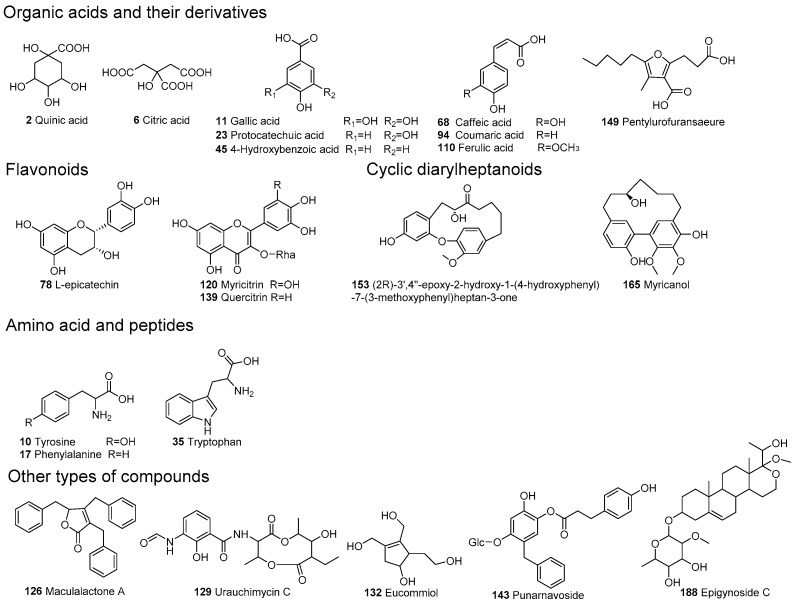
Chemical structures of some constituents identified in the extract of *Myrica rubra*.

The precise mass of the precursor ions from the MS^1^spectra and product ions from MS^2^ spectra can meet regulatory requests for qualitative analysis, and the combinations of both have been extensively used in the analysis of complex samples [28,29,30,31,32,33]. A total number of 188 compounds were detected in the extract of *Myrica rubra* leaf (listed in Supplementary Materials Table S1), and the total ions current (TIC) chromatograms by UPLC-HRMS were displayed with negative and positive modes in Figure 2A,B, respectively. Chemical structures of some identified compounds are depicted in Figure 3, including organic acids and their derivatives, flavonoids, cyclic diarylheptanoids, amino acids and peptides etc. Among them, eight compounds (seen in Section 3.3) were explicitly identified by comparison of their retention times and MS spectra with the reference substances. By analyzing the molecular formula and MS^2^ fragmentation information, another 108 compounds were characterized tentatively with the aid of the Chemical Abstracts database. A mass error of less than 6 ppm was routinely achieved for the accurate mass measurement.

#### 2.1.1. Identification of Organic Acids and Their Derivatives

Twenty four organic acids and their derivatives were detected in the extract of *Myrica rubra* leaf. They were only detected in negative ion mode for the presence of carboxyl, so further analysis and discussion were employed in this mode. Among them, compounds **11**, **23**, **45** and **68** were explicitly verified to be gallic acid, protocatechuic acid, 4-hydroxybenzoic acid, and caffeic acid in accordance with reference standards. They all produced their characteristic fragment ions by the neutral loss of CO_2_, which suggested the existence of a carboxyl group in their molecular structures [34].

Compound **51**demonstrated a [M − H]^−^ ion at *m*/*z* 183.02815 for an elemental composition of C_8_H_7_O_5_, which was 15 Da more than that of gallic acid (compound **11**, *m*/*z* 169), implying the pesence of a methyl group attached to gallic acid. Therefore, compound **51** was deduced to be methylgallic acid. Compounds **20**, **42** and **49** were tentatively ascribed to be dihydroxybenzoic acids for all of them shared the identical quasi-molecular ions and fragment routes with protocatechuic acid (compound **23**). Compound **38** was xyloside for it generated an aglycone ion at *m*/*z* 153 by the loss of xylosyl, followed by the same fragmentation pathway. Compound **110** was observed as a deprotonated molecule at *m*/*z* 193 in negative ion mode, which was 14 Da (CH_2_) more than that of caffeic acid (compound **68**), indicative of a methyl group connected to it. By searching the compounds reported in natural products, compound **110** was inferred to be ferulic acid. Meanwhile, compound **94** generated a precursor ion at *m*/*z* 163 (C_9_H_7_O_3_), which was 16 Da less than that of caffeic acid, implying there were only one hydroxyl in the benzene ring, so it was assumed to be coumaric acid. Furthermore, compound **61** was identified as its glucoside due to the occurrence of fragmentation ions at *m*/*z* 163 [M – H − glucosyl]^−^ and *m*/*z* 119 [M – H – glucosyl − CO_2_]^−^.

#### 2.1.2. Identification of Flavonoids

**Figure 4:**
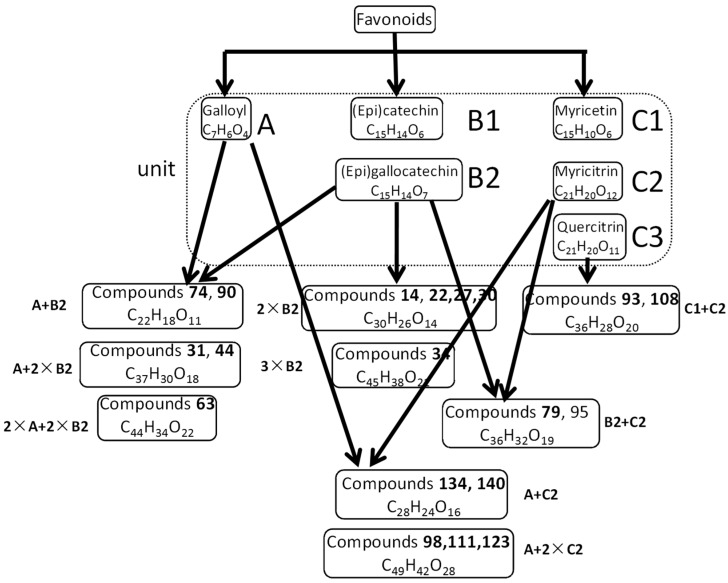
Identification of flavonoids isolated from *Myrica rubra* leaf extract according to their structure units.

There were three types of flavonoids identified in the extract of *Myrica rubra*, namely flavan-3-ols, flavonols and xanthones. Their UV spectra and fragmentation pathways bear similarities and differences, which would be helpful for their identification and differentiation. The identification of this type of compounds is partly displayed in Figure 4.

##### Flavan-3-ols

Investigations on flavan-3-ols or polyphenols have gained significant attention for their remarkable activities and wide existence in the plant kingdom [9]. Three distinctive fragmentation patterns are mainly recognized as Retro-Diels-Alder fission (RDA), quininemethide fission (QM), and heterocyclic ring fission (HRF) [35].

Compound **78** was unequivocally ascertained to be L-epicatechin according to comparison with an authentic standard. In the MS^2^ spectrum, the [M − H]^−^ ion at *m*/*z* 289.07062 gave the base peak ion at *m*/*z* 247 by the loss of C_2_H_2_O. In addition, it produced a product ion of high abundance at *m*/*z* 125 through the cleavage of A^1,4^ type, along with the B ring fragment ion at *m*/*z* 109. In positive ion mode, the [M + H]^+^ ion gave an A^1,3^ ion at *m*/*z* 139 as the base peak from RDA reaction. There were several subclasses of compounds of this type (shown in Figure 3).

Compounds **24** and **53** had identical quasi-molecular ions at *m*/*z* 307 (C_15_H_13_O_7_) in negative ion mode, which was 16 Da more than that of L-epicatechin, indicating that there was a hydroxyl substituent. Moreover, daughter ions at *m*/*z* 125 and 109 could serve as diagnostic ions to presume them to be (epi)gallocatechin. Another series of isomers, compounds **22**, **27** and **30**, could be deduced as (epi)gallocatechin-(epi)gallocatechin, which displayed a [M − H]^−^ ion at *m*/*z* 609, corresponding to dimer of (epi)gallocatechin. In analogy, compound **34** was presumed to be (epi)gallocatechin-(epi)gallocatechin-(epi)gallocatechin, a trimer of (epi)gallocatechin, based on precisemass measurements and MS^2^ fragmentation patterns.

For compounds **74** and **90**, deprotonated molecules at *m*/*z* 457 underwent similar fragment pathways as compounds the mentioned above for their common product ions. The base peak ion at *m*/*z* 169 (C_7_H_5_O_5_) in MS^2^ spectra suggested the presence of a galloyl moiety in the molecular structure in negative mode. Consequently, they were tentatively characterized as (epi)gallocatechingallate. Similarly, compound **63** was plausibly inferred to be (epi)gallocatechingallate-(epi)gallocatechingallate, a dimer of (epi)gallocatechingallate. The different fragment pathways between compound **63** and **34**, as well as their identification were verified in previous reports [10]. Compounds **31** and **44** were assigned as (epi)gallocatechin-(epi)gallocatechingallate, which combined a unit of (epi)gallocatechin (C_15_H_14_O_7_) and (epi)gallocatechingallate (C_22_H_18_O_11_) and underwent similar fragmentation pathways as those described in the literature [9,36].

##### Flavonols

Flavonols were also detected in the extract of *Myrica rubra* leaf. These compounds produced abundant fragment ions in both positive and negative ion mode, while the ion response of the latter one was better. As a result, we only discuss the fragmentation pathways observed in negative ion mode.

One main structure unit of this type was myricitrin (compound **120**) [37], which was explicitly characterized by comparison with a reference sample. The fragment ion at *m*/*z* 316 in the MS^2^ spectrum was generated by the loss of a rhamnosyl residue (146 Da), and further yielded characteristic ions at *m*/*z* 151 and 179 owing to cleavages of the A^1,3^ and B^0,3^ type, which are often shown in flavonols [33]. Compound **72** exhibited a pseudomolecular ion [M − H]^−^ at *m*/*z* 915.16467 (C_42_H_37_O_24_), whose MS^2^ fragmentation information resembled that of compound **120**. The molecular formula was a double confirmation to ascribe it to myricitrinyl-myricitrin.

Compounds **134** and **140** gave rise to identical deprotonated molecules at *m*/*z* 615, which fragmented to [M − H − galloyl-rhamnoyl]^−^ ion at *m*/*z* 371 with high abundance, implying the existence of myricitrin. The product ion at *m*/*z* 169 in MS^2^ spectrum also indicated the presence of a gallic acid fragment in the structure. According to the chemical constituents reported in [10,38], these two compounds were tentatively deduced to be myricitringallate.

From all the characterization discussed above, it’s not difficult to find the composition of flavonoids in the extract of *Myrica rubra* leaf. The units were gallic acid (C_7_H_6_O_4_), (epi)catechin (C_15_H_14_O_6_), (epi)gallocatechin (C_15_H_14_O_7_), and myricitrin (C_21_H_20_O_12_) or myricetin (C_15_H_10_O_8_), which composed various compounds, including several potential new ones. Taking compounds **54**, **79**, **95** and **107** for example, they shared the same deprotonated molecules at *m*/*z* 767 in negative ion mode and molecular formula C_36_H_32_O_19_ given by high resolution mass spectra. Their UV spectra shapes were alike, with λ_max_ at 270 nm and they appeared to belong to proanthocyanidins [10]. Typical fragment ions at *m*/*z* 125, 151, 169, and 179 were observed in the MS^2^ spectra in accordance with what we summarized previously. Thus they were deduced to contain one (epi)gallocatechin and one myricitrin moiety in their molecular structures, and were named as myricitrinyl-(epi)gallocatechin. Another pair of isomers belonging to this type were compounds **93** and **108** according to their consistent fragmentation pathways. By analyzing the molecular formula (C_36_H_28_O_20_) obtained from HRMS, we inferred the extract comprised a molecule of myricetin and a molecule of myricitrin, although their linkage position remained uncertain. Additionally, some gallates esterified with myricitrinyl-myritrin were also identified in the extract, i.e., compounds **98**, **111** and **123**. As all these compounds couldn’t be found in the Chemical Abstracts database, they may be novel compounds.

The other primary structure unit of this type was quercitrin (compound **139**), which displayed a [M − H]^−^ ion at *m*/*z* 447 in negative ion mode, resulting in a base peak ion at *m*/*z* 300 and another dominant ion at *m*/*z* 301 by eliminating a rhamnosyl unit, and the fragment behavior of aglycone was similar to that of myricitrin. Both compounds **146** and **150** exhibited a deprotonated molecular ion at *m*/*z* 489, which was 42 Da (C_2_H_2_O) higher than that of compound **139** and their MS^2^ spectra were homologous, implying that an acetyl group was linked to the quercitrin, however the position of the linkage was still not determined, so they were provisionally presumed to be acetylquercitrin. Compound **136** was identified as quercitrinyl-(epi)gallocatechin methyl gallate in a similar way.

Moreover, a range of new compounds that haven’t been reported previously were discovered by means of permutations and combinations of the aforementioned units. Compound **114** showed a molecular formula of C_41_H_36_O_23_, producing a base peak ion at *m*/*z* 301 (C_15_H_10_O_7_), together with other diagnostic ions at *m*/*z* 125, 151 and 179, in its MS^2^ spectrum, thus it’s speculated that a quercitrin (C_21_H_20_O_11_) group connected to another (C_20_H_18_O_12_) unit existed in its molecular structure. By searching the Chemical Abstracts database, C_20_H_18_O_12_ may be quercetin-galactoside, another flavonol of the same aglycone. As a result, compound **114** was plausibly characterized as quercitrinyl-quercetin-galactoside. Homoplastically, compounds **104** and **109** were assigned to be consisting of myricitrin and quercetin-arabinoside.

##### Xanthones

Only compound **119** was identified as a xanthone. It yielded a dominant ion as the base peak at *m*/*z* 125 after the breakdown of C1-4 in the MS^2^ spectrum, indicating the existence of two substituent groups in the B ring. Other fragment ions at *m*/*z* 259, 243 and 223 corresponded to the characteristic losses of CO, CO_2_, and 2 × CH_4_O. Consequently, compound **119** was ascribed to be dimethoxy-dihydroxyxanthone with an as yet unknown linkage form.

#### 2.1.3. Identification of Cyclic Diarylheptanoids

Cyclic diarylheptanoids are characteristic and representative components in *Myrica rubra* [39,40,41], showing clean and abundant mass spectra in positive ion mode. Their mass spectrometry fragmentation patterns have rarely been reported, so we tried to discuss them in this study. Compounds **153** and **165** were positively identified as (2*R*)-3′,4′′-epoxy-2-hydroxy-1-(4-hydroxy-phenyl)-7-(3-methoxyphenyl)heptan-3-one and myricanol according to the retention time and MS spectra comparison with authentic standards.

The quasi-molecular ion [M + H]^+^ of compound **153** experienced a series of cleavages and rearrangements, and formed fragment ions of moderate abundance at *m*/*z* 107 and 123, which were attributed to a methyl substituted phenol residue and a methyl substituted resorcinol residue, respectively. On the other hand, the elimination of a diaryl moiety led to the base peak ion at *m*/*z* 131 in the MS^2^ spectrum.

In the positive ion mode MS spectrum compound **165** showed a remarkable protonated [M + H]^+^ molecule at *m*/*z* 359, and produced a base peak ion at *m*/*z* 271 by the loss of a pentanol group. Other fragment ions at *m*/*z* 259 and 341 originated from the elimination of an octanol moiety and H_2_O. Moreover, a weaker ion at *m*/*z* 211 occurred after the cleavage of the C1-2 bond followed by the loss of a molecule of C_9_H_8_O_2_.

#### 2.1.4. Identification of Amino Acids and Peptides

Amino acids and peptides are widespread in natural products, showing various biological activities such as antimicrobial, anticancer, anti-cardiovascular disease effects and so on. There were four amino acids containing primary amines, five amino acids containing secondary amines, and three peptides detected. The primary amino acids yield protonated [M + H]^+^ molecules and subsequently underwent successive losses of several NH_3_, H_2_O and CO_2_ groups. However, compounds **85** and **91**, secondary amino acid isomers, preferentially lost a molecule of methacrylic acid (C_4_H_6_O_2_) to form the base peak ions at *m*/*z* 130. Based on the MS^n^ fragment information and high resolution mass spectrometry, they could be tentatively identified as monascumic acid or its isomers. Compounds **76**, **83** and **102** showed a pseudomolecular ion [M + H]^+^ at *m*/*z* 409 of which the molecular formula was C_16_H_28_O_10_N_2_. They tended to eliminate the C_6_H_13_NO_5_ group to afford a fragment ion at *m*/*z* 230, ascribed to be *N*-(1-deoxyfructopyranos-1-yl)isoleucyl aspartic acid or its isomers. For the peptides, their MS^2^ fragmentation spectra gave fragment ions by the cleavage of peptide bonds except the neutral loss of NH_3_, H_2_O and CO_2_, which were helpful for the prediction.

#### 2.1.5. Identification of Other Types of Constituents

There were also other types of micro-constituents detected in the extract of *Myrica rubra*, including naphthoquinones, terpenoids, polysaccharides, steroids, etc. For compounds **37** and **69**, preliminary mass spectra performed in TIC mode showed [M − H]^−^ ions at *m*/*z* 451 in positive ion mode. Then they generated a series of product ions of glycosyl segments depending on the voltage applied to the source, such as ions at *m*/*z* 59, 89, 101, and 119. Another pentyl moiety daughter ion was observed at *m*/*z* 71. Finally, their structures were established as glucosyl-octenyl-glucose. However, it was arduous to distinguish the linkage position between the octenyl units and the glucosyl group. In a similar way, compounds **75**, **84** and **97** were tentatively assigned as glucosyl-hexanoyl-glucose.

Compounds **64** and **70** showed a pseudomolecular ion [M − H]^−^ at *m*/*z* 447 with fragment ions at *m*/*z* 59, 89, 101, and 161, which may be due to the existence of a glycosyl group. An additional product ion at *m*/*z* 71 corresponded to the successive loss of an oxygen heterocycle and acetic acid. By searching the Chemical Abstracts database, they were deduced to be *O*-acetylshanzhiside methyl ester or its isomers, which are identified herein in the *Myrica* genus for the first time. The predominant fragment ions of compounds **73**, **81** and **101** were rather similar, except for the molecular weight of 16 Da less, so probably the *O*-acetyl group was replaced by a propyl. Consequently, the three isomers were tentatively identified as propyl shanzhiside methyl ester or its isomers, although the linkage position still could not be determined, which might be novel compounds.

### 2.2. Effects of the Whole Extract and Typical Compounds on H_2_O_2_-Induced Changes in N2a Cells

#### 2.2.1. Effects on H_2_O_2_ Induced Cell Death by MTT Assays

**Figure 5:**
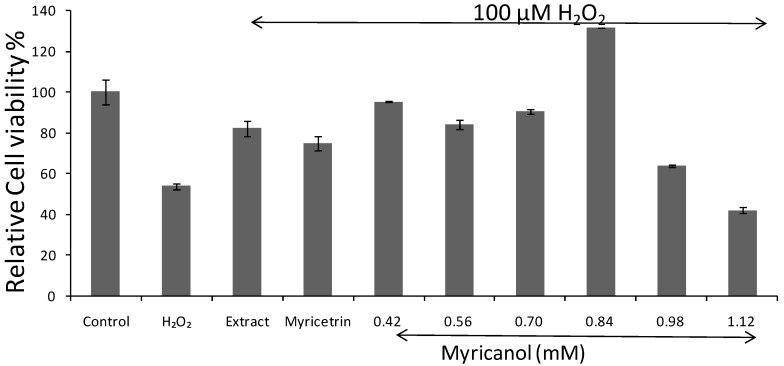
Effect of the whole extract (20 μg/mL), myricetrin (0.65 mM) and myricanol at various concentrations on H_2_O_2_-induced oxidative stress in N2a cells by MTT assay.

To establish the conditions for the research, the effect of H_2_O_2_ and serum on N2a cells were investigated. Cells incubated with or without serum medium both displayed a H_2_O_2_-induced anti-cell proliferation at the concentration of 50–100 μM. For the medium supplemented with serum, the concentration of H_2_O_2_ did not demonstrate significant difference, and 100 μM was chosen for the latter trail. Cell viability was measured by MTT reduction assay. Results were means ± S.D. from three independent experiments. As shown in Figure 5, exposure of N2a cells to H_2_O_2_ 100 μM alone sharply reduced the cell viability (approximately 50% of the control group value). The anti-cell proliferation was dramatically attenuated by pretreatment of N2a cells with myricanol at various concentrations without cytotoxicity, and the relative cell viability came to the highest at the concentration of 0.84 mM. Besides, pretreatment with the whole extract of *Myrica rubra* leaf at the concentration of 20 μg/mL, followed by 100 μM H_2_O_2_ for 8 h, it suppressed the reduction in relative cell viability (82.16 ± 4.15%). Pretreatment of N2a cells with myricetrin altered the relative viability (74.60 ± 3.45%) at the highest non-toxic concentration (0.65 mM). The result demonstrated a protective effect of myricanol against H_2_O_2_-induced cytotoxicity.

#### 2.2.2. Effects on H_2_O_2_ Altered Cell Morphology

**Figure 6:**
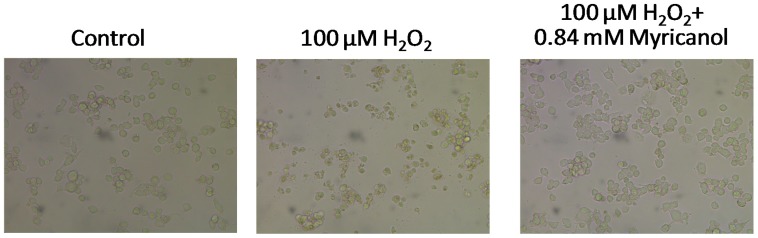
Effect of myricanol on H_2_O_2_-induced cell morphological changes.

Control cells with good growth were plump and intact. Upon exposure to 100 μM H_2_O_2_, N2a cells floated in the culture media and appeared round, with completely altered neuronal outgrowths, indicative of the cytotoxicity of H_2_O_2_. In H_2_O_2_+myricanol group, there were noticeably fewer damaged and floating cells compared with the H_2_O_2_ group, cells showed healthy morphology similar to that of untreated control group (Figure 6). This confirmed the protective effect of myricanol on H_2_O_2_-induced cell damage and was consistent with the MTT assay result.

#### 2.2.3. Effects on H_2_O_2_-Induced Intracellular ROS

**Figure 7:**
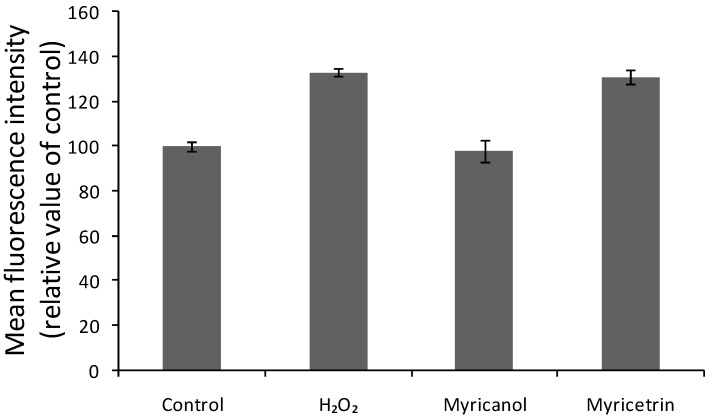
Effect of myricanol and myricetrin on intracellular ROS in N2a cells. Myricanol and myricetrin at the concentration of 0.84 mM and 0.65 mM, respectively.

Oxidative stress is thought to be induced by excess of ROS, which may be byproducts of cellular metabolism and could be quantified by the fluorescent probe 2′,7′-dichlorofluorescin diacetate (DCFH-DA). DCFH can be oxidized to a highly fluorescent DCF, indicating the resultant oxidative stress due to overproduction of ROS [42]. N2a cells exposed to H_2_O_2_ alone with myricetrin displayed a significant increase in DCF fluorescence, while when pretreated with myricanol at a concentration of 0.84 mM, the DCF signal was attenuated compared with the H_2_O_2_ group (shown in Figure 7). In addition, myricetrin did not exert significant protective effect.

#### 2.2.4. Effects on H_2_O_2_-Induced Intracellular Calcium Concentration

It had been widely reported that calcium invokes many Ca^2+^-dependent enzymes which play a remarkable role in regulating various cellular components, while the excessive entry of Ca^2+^ may cause neurotoxicity through cytoplasmic and nuclear processes [43,44]. In the study, we estimated the effect of two compounds on cytosolic Ca^2+^ shifts, originating from both extra and intracellular Ca^2+^ sources. H_2_O_2_ was reported to induce biphasic elevation of [Ca^2+^]i, resulting in disruption of cytosolic calcium homeostasis [45]. In this study, [Ca^2+^]i increased to 3045.51 ± 572.69 (*p* < 0.05) upon cells’ exposure to 100 μM H_2_O_2_ (Table 1). However, when pretreated with myricanol, no obvious increase in cytosolic Ca^2+^ was observed (777.81 ± 23.49). The Ca^2+^ shifts were only moderate when pretreated with myricetrin by contrast (1178.92 ± 106.93). This observation further verified the protective effects of myricanol against H_2_O_2_-induced cytotoxicity via relieving [Ca^2+^]i overload in N2a cells.

### 2.3. Discussion

UPLC-PDA-HRMS was used for the identification of chemicals in the extract of *Myrica rubra* leaf. One hundred and sixteen compounds were firmly or tentatively identified, among which there were 24 pairs of isomers, whose detailed structures could not be determined via mass spectrometry and need the aid of nuclear magnetic resonance. What’s more, we only studied the components of the high polarity fraction, leaving the low polar one which may contain abundant constituents and be a complement for the overall chemical profile displayed by *Myrica rubra*.

Neurodegenerative disorders have been linked to oxidative stress, which may contribute to the generation of ROS [46,47]. H_2_O_2_ is often used as neurotoxin to induce oxidative stress and damage in neuronal cells via production of ROS [24] and overload of calcium ions [48]. The cytotoxicity of H_2_O_2_ to N2a cells was proved in this study by MTT assays of cell viability and morphological observation (Figure 4 and Figure 5). The cells pretreated myricanol showed significantly increased cell viability, and the cell morphology resembled that of the control groups even after exposure to H_2_O_2_, suggesting its protective role in H_2_O_2_-induced oxidative stress. The generation of ROS and the shift of [Ca^2+^]i were determined by the fluorescent DCFHDA probe and Fura 2-AM probe, respectively. The increase of ROS induced by H_2_O_2_ was markedly attenuated by the myricanol treatment. Little elevation of [Ca^2+^]i was observed when pretreated with myricanol compared with H_2_O_2_ group.

The investigation clearly revealed that myricanol inhibited H_2_O_2_-induced neuronal death, ROS generation and [Ca^2+^]i overload. Myricanol is a typical cyclic diarylheptanoid containing two phenolic hydroxyl groups and one alcoholic hydroxyl group, which may lead to powerful ROS scavenging properties and neuroprotective activities. Myricitrin is a typical flavonoid, also containing numerous phenolic hydroxyl groups, however, it demonstrated insignificant pharmacological activity. This was only a preliminary investigation of the neuroprotective effects of myricanol. Relevant aspects (cell viability, ROS production and intracellular Ca^2+^) leading to the stress condition merit further research, as well as the difference of the structure and neuroprotective mechanism between these compounds. This work may also provide experimental platform for further research in human cell lines to test the neuroprotective activity.

## 3. Experimental Section

### 3.1. Chemicals, Reagents and Materials

*Myrica rubra* leaves were collected from Wenzhou Lon-Yang Agricultural Integrative Development Company (Wenzhou, China). The plant materials were confirmed by one of the authors (C.H. Yang), and a sample specimen (No. YM0001) had been deposited in the Key Laboratory for Dao-Di Herbs Biotechnology of Fujian Province, at Xiamen Medical College in Xiamen, China.

HPLC-grade acetonitrile (Merck, Darmstadt, Germany) and formic acid (ROE Scientific Inc., Newark, NJ, USA) were used for UPLC analysis. Deionized water was purified by a Milli-Q system (Millipore, Molsheim, France). Other reagents and chemicals used for the preparation and separation of the whole extract were analytical grade.

MTT, L-Glutamine, DCFH-DA, dimethylsulfoxide (DMSO), along with reference substances of gallic acid (**11**), protocatechuic acid (**23**) and 4-hydroxybenzoic acid (**45**) was purchased from Sigma-Aldrich (Saint Louis, MO, USA). L-Epicatechin (**78**) was acquired from Chengdu Herbpurify. Co., Ltd (Chengdu, China). Fura2-AM was obtained from DojinDO Molecular Technologies, Inc. (Kumamoto, Japan).

N2a cells were obtained from the cellbank of the Chinese Academy of Sciences (CAS, Shanghai, China). Dulbecco’s Minimal Essential medium (DMEM) HIGH GLUCOSE was purchased from HyClone (Logan, UT, USA) and Fetal Bovine Serum (FBS) was from Gibco BRL (Grand Island, NY, USA). The Fluo-4 NW Calcium Assay Kit was from Thermo Fisher Scientific, Inc. (Bremen, Germany).

### 3.2. Apparatus and Chromatographic Conditions

An Acquity^TM^ UPLC system (Waters, Milford, MA, USA) was used to perform the separations using water containing 0.1% formic acid (phase A) and acetonitrile (phase B) as LC solvents, and a InertSustain C18 column (2.1 × 75 mm, 2 μm; GL Sciences Inc., Tokyo, Japan). The flow rate was 0.2 mL·min^−1^. After injection of 0.1 μL samples, a 50 min elution at 30 °C was applied with a linear gradient as follows: 0 min, 3% B; 25 min, 50% B; 35 min, 100% B; 50 min, 100% B. The photo-diode array (PDA) detector performed the wavelength scanning from 190 nm to 400 nm.

High resolution MS analysis was recorded on a Q Exactive quadrupole Orbitrap mass spectrometer (Thermo, Bremen, Germany) coupled with an ESI source in both negative and positive mode. Data were acquired by Higher Energy Collision Induced Dissociation. The full scan-ddMS2 mode was applied with the optimized MS parameters set as: spray voltage 2.5 kV for ESI^−^ and 3.5 kV for ESI^+^; sheath gas 49 arb; Aux gas 12 arb; probe heater temperature 420 °C; capillary temperature 260 °C; S-lens RF level 50; scan rang 100–1500 Da; resolution 70 k FWHM (at *m*/*z* 200); HCD fragmentation energy 30%. MS data collected were processed utilizing Thermo Scientific^TM^ Xcalibur^TM^ platform (San Jose, CA, USA).

### 3.3. Preparation of Standard Solutions and Samples

The leaves of *Myrica rubra* (ca. 10 kg) were ground and extracted with boiling water (20 L × 3). A small portion (100 mL) of the whole extracts was concentrated to yield a dark-brown powder (32.1 mg), which was then dissolved in 1 mL water for UPLC-HRMS analysis after filtration through a 0.22 μm membrane. The remaining extract was rinsed with methanol for several times on a Sephadex LH-20 column into 20 portions. Subsequently, eluates of lower polarity were passed over a Waters Sep-Pak SPE column (Waters Corp., Milford, MA, USA) with gradient profile of acetone/*n*-hexane at the proportion of 1:24, 1:19, 1:12, 1:9, 1:3,1:2 and 1:1, followed by pure acetone. Thus eight components were obtained according to the polarity, and the middle and high polarity ones were fractionated by TBE-300B high-speed countercurrent chromatography. The solvent system consisting of hexane/ethyl acetate/methanol/water (1:1:1:1), the portion of medium polarity were further subjected to preparative HPLC (Interchim^®^ puriFlash 4250) to yield 4 compounds with linear gradient methanol (10–100%) in 50 min. Myricanol and (2*R*)-3′,4′′-epoxy-2-hydroxy-1-(4-hydroxyphenyl)-7-(3-methoxyphenyl)heptan-3-one was obtained from the 72% methanol wash, while the 34% methanol wash generated myricitrin and caffeic acid.

For qualitative analysis, standard solution containing eight reference substances with known concentrations (10 μg/mL dissolved in methanol) was prepared and stored at 4 °C until use. The eight reference substances were gallic acid (**11**), protocatechuic acid (**23**), 4-hydroxybenzoic acid (**45**), caffeic acid (**68**), L-epicatechin (**78**), myricitrin (**120**), (2*R*)-3′,4′′-epoxy-2-hydroxy-1-(4-hydroxy-phenyl)-7-(3-methoxyphenyl)heptan-3-one (**153**) and myricanol (**165**).

For evaluation of neuroprotective activity, 1 mg powder of myricanol and myricitrin were dissolved in DMSO to a concentration of 50 mg/mL. Fifty mg of freeze-dried powder of the whole extract was dissolved in 1 mL DMSO.

### 3.4. Evaluation of Neuroprotective Effects against H_2_O_2_-Induced Changes in N2a Cells

#### 3.4.1. Cell Culture

N2a cells were maintained in high-glucose DMEM containing 10% FBS and 2 mM L-glutamine in an atmosphere of 95% relative humidity (5% CO_2_) at 37 °C. The media was changed every other day.

#### 3.4.2. Analysis of Cell Viability by MTT Assay

Cells seeded into 96-well microplate (2×10^4^ cells/well) were challenged with 100 μM H_2_O_2_ to induce oxidative stress. For administration groups, cells were pretreated with various non-cytotoxic concentrations of myricanol and myricetrin for 12 h (not withdraw before adding H_2_O_2_). After 8 h’s incubation with H_2_O_2_, 10 μL of 5% MTT was added into the medium. Following 2–4 h incubation, the medium was discarded and replaced by 10 mL acidified isopropyl alcohol. Then cell viability was evaluated in an ELISA reader (INFINITE M1000PRO, Tecan US, Morrisville, NC, USA) by measuring the optical densities at 570 nm. For the control group, cell cultures were incubated with the same culture medium of equal volume. The culture medium containing 10 μL of 5% MTT was adopted as the blank group. The viability (%) was calculated with the following formula:

$$\mathrm{Viability}\;\%=\frac{\mathrm{Average}\;\mathrm{of}\;\mathrm{test}\;\mathrm{wells}\;O.D.\;-\;\mathrm{Average}\;\mathrm{of}\;\mathrm{blank}\;\mathrm{wells}\;O.D.}{\mathrm{Average}\;\mathrm{of}\;\mathrm{control}\;\mathrm{wells}\;O.D.\;-\;\mathrm{Average}\;\mathrm{of}\;\mathrm{blank}\;\mathrm{wells}\;O.D}\times 100\%$$

#### 3.4.3. Observation of Cellular Morphology

According to aforementioned procedure of Section 3.4.2, N2a cells were cultured in 96-well microplate (2 × 10^4^ cells/well) and exposed to 100 μM H_2_O_2_ for 8 h. For the trial, myricanol and myricetrin isolated from *Myrica rubra* were added in advance to assess their neuroprotective effect. The observation and visualization of cellular morphology were conducted by an inverted microscope (Leica, Malvern, PA, USA) equipped with a DF450C system.

#### 3.4.4. Measurement of ROS Production

According to the aforementioned procedure described in Section 3.4.2, N2a cells were cultured in 96-well microplate (2 × 10^4^ cells/well) and exposed to 100 μM H_2_O_2_ for 8 h. For the trial, N2a cells were pretreated with myricanol and myricetrin for 12 h, followed by treatment with 10 μM DCFH-DA for 30 min. The solution was discarded and washed with PBS for another three times. 100 μL media was added into the microplate. The generation of ROS was determined by fluorimetric detection with an ELISA reader (INFINITE M1000PRO, Tecan US, Morrisville, NC, USA) at an excitation–emission wavelength of 485–538 nm respectively. Alteration of ROS was calculated by the DCF fluorescence intensity in contrast with the control group.

#### 3.4.5. Measurement of Intracellular Calcium Concentration

According to the procedure described in Section 3.4.2, N2a cells were cultured in 96-well microplates (2 × 10^4^ cells/well) and exposed to 100 μM H_2_O_2_ for 8 h. For the trial, N2a cells were pretreated with myricanol and myricetrin for 12 h. The media were discarded, and washed with PBS for twice, followed by treatment with 5 μM Fura-2-AM for 30 min at 37°C. The solution was discarded and washed with PBS for another three times. 100 μL media was added into the microplate and the concentration of [Ca^2+^]i was detected with a ELISA reader (INFINITE M1000PRO, Tecan US, Morrisville, NC, USA). Triton-X 100 was used for disruption of the cell membrane, and EGTA was used for the complexation of calcium. On binding Ca^2+^, the excitation wavelength shifted to 340 and 380 nm, while the emission wavelength remained at 510 nm. The concentration of calcium was calculated with the following formula: [Ca^2+^]i = K_d_(F_0_−F_min_)/(F_max_−F_0_), where K_d_ (assuming it as 224 nM) was the dissociation constant of the chemical reaction for Ca^2+^ buffering by the fluorescent dye. F_0_, F_max_ and F_min_ stand for the fluorescence measured without adding Triton-X 100 or EGTA, with adding 0.1% Triton-X 100 and by quenching Fluo-3 fluorescence with 5 mM EGTA, respectively.

### 3.5. Statistical Analysis

Except for where stated otherwise, all results were expressed as the mean ± standard deviation (SD) of the indicated measurements of quintuplicate experiment. The significance of differences was determined by Student’s *t*-test, and a *p* value less than 0.05 was considered statistically significant. All data were analyzed using InStat3 or Prism software 5.0b (GraphPad Software, San Diego, CA, USA).

## 4. Conclusions

In this research, UPLC coupled with Q Exactive quadrupole Orbitrap mass spectrometry was applied to identify the chemical constituents in the extract of *Myrica rubra* leaf. The chemical profile was comprehensively and systematically studied for the first time. By comparison with authentic substances, integration of exact mass, UV spectra, and fragment information, 116 compounds were confirmed or tentatively identified, including 26 organic acids and their derivatives, 36 flavonoids, two cyclic diarylheptanoids, 12 amino acids and peptides, together with 40 other types of compounds. Each category was analyzed in detail to summarize and conclude available fragmentation rules in HRMS. In particular, a convenient and effective strategy was proposed for the rapid characterization of flavonoids through permutation and combination of molecular formula, which were comprised of specific units, i.e., galloyl, (epi)catechin, (epi)gallocatechin, myricetin, myricitrin, and quercitrin.

The neuroprotective activities of two representative components, myricetrin and myricanol (one flavonoid and one cyclic diarylheptanoid, respectively) were evaluated onH_2_O_2_-inducedN2a cells by MTT assays. The results revealed that a significant cell death trigged by H_2_O_2_ was neutralized by myricanol, while myricetrin only had moderate effect. Further confirmation was conducted by intracellular ROS and calcium ion assays, for the decrease in ROS production and Ca^2+^ shifts were both observed. In summary, myricanol might offer a promising the rapeutic strategy to reduce the neurotoxicity of reactive dicarbonyl compounds, providing a potential benefit agent with age-related neurodegenerative diseases.

## Figures and Tables

**Table 1:** Effect of myricanol and myricetrin on intracellular calcium shifts determined by Fura-2-AM probe.

Group	Concentration	Concentration of Intracellular Calcium/nM
Control	-	611.60 ± 33.81
H_2_O_2_	100 μM	3045.51 ± 572.69
Myricanol + H_2_O_2_	0.84 mM + 100 μM	777.81 ± 23.49
Myricetrin + H_2_O_2_	0.65 mM + 100 μM	1178.92 ± 106.93

## Supplementary Materials

Supplementary materials are available online.
