# Supplementary material for: Study on Chemical Profile and Neuroprotective Activity of Myrica rubra Leaf Extract
Source: Molecules. 2017 Jul 24;22(7):1226. doi: 10.3390/molecules22071226 (PMC6152229; doi:10.3390/molecules22071226)
Supplement: Supplementary file 1 [file molecules-22-01226-s001.pdf]

Table S1. Characterization of constituents in the extract of *Myrica rubra* by UPLC-HRMS

No.	t/min	Formula	ESI-MS(-) Mean Measured Mass (m/z)	ESI-MS(-) Error (ppm)	ESI-MS(+) Mean Measured Mass (m/z)	ESI-MS(+) Error (ppm)	Identification	MS2
1	1.74	C13H24O12	371.11780	-1.623			Glyceromannoheptoyl-mannose	MS2[371@30]:191(100),179(4)
2	1.87	C7H12O6	191.05421	-4.211			Quinic acid	MS2[191@30]: 191(100),147(1),111(1),93(5),85(15),59(1)
3	2.04	C23H18O7			407.11517	6.486	Unidentified	MS2[407@30]: 215(100)
4	2.48	C4H9O4N			136.06157	8.347	Hydroxythreonine	MS2[136@30]: 119(1),101(1)
5	2.66	C9H7O3N6			247.05724	-0.707	Unidentified	MS2[247@30]: 147(100),189(90)
6	2.70	C6H8O7	191.01940	4.141			Citric acid	MS2[191@30]: 57(4),85(30),87(42),111(100),129(7)
7	3.00		405.02664	6.251	407.04236	6.392	Unidentified	MS2[405@30]: 85(10),87(19),111(100),129(8),191(46)<br>MS2[407@30]: 215(100)
8	3.40	C9H18O8	253.09167	-0.49			Hexosylglycerol	MS2[253@30]: 62(78),75(5),117(31),180(11),191(100)
9	3.43				247.05724	-1.112	Unidentified	MS2[247@30]: 86(48),113(100),147(8),189(8)

Table S1. *Cont*.

10	4.03	C9H11O3N			182.08076	-2.25	Tyrosine	MS2[182@30]:119(13),123(27),136(100),137(10),147(20),165(55),166(6)
11*	5.50	C7H6O5	169.01230	-5.028	171.0284	-2.338	Gallic acid	MS2[169@30]:125(100),126(7)
12	5.65		779.14288				Unidentified	MS2[779@30]:83(31),125(68),137(32),167(100),179(9),383(5),661(6)
13	5.75				215.05214		Unidentified	MS2[215@30]:72(63),125(8),195(16),197(13)
14	5.87	C30H26O14			611.13873	-1.312	(Epi)gallocatechin-(epi)gallo-catechin	MS2[611@30]:127(3),139(93),163(100),257(6),275(26),407(5)
15	6.02	C11H14O5			227.09087	-2.334	Veratroyl ethylene glycol or its isomers	MS2[227@30]:121(10),149(18),167(100),177(37),209(8)
16	6.02				267.08325		Unidentified	MS2[267@30]:136(100)
17	6.62	C9H11O2N			166.08583	-2.56	Phenylalanine	MS2[166@30]:120(100),131(5)
18	6.85				287.10944		Unidentified	MS2[287@30]:153(10)
19	7.40				319.04367		Unidentified	MS2[319@30]:72(8),86(14),153(9),157(73),185(15),203(16),245(10),263(6),273(30)
20	7.55	C7H6O4	153.01755	-4.478			Dihydroxybenzoic acid	MS2[153@30]:125(3),109(4)
21	8.43		261.00641				Unidentified	MS2[261@30]:119(23),181(100)
22	8.52	C30H26O14	609.12268	-1.973	611.13922	-0.51	(Epi)gallocatechin-(epi)gallo-catechin	MS2[611@30]:127(90),139(100),163(4),179(42),275(23),287(45),425(5)
23*	8.92	C7H6O4	153.01740	-5.458			Protocatechuic acid	MS2[153@30]:109(100)

Table S1. *Cont*.

24	9.08	C15H14O7	305.06546	-0.391	307.08047	-2.472	(Epi)gallocatechin	MS2[305@30]:109(10),125(100),137(20),179(14),167(19)
25	9.32				355.09918		Unidentified	MS2[355@30]:185(20),192(60),193(51),324(12)
26	9.77				267.15613		Unidentified	MS2[267@30]:223(18),239(33),241(45),259(15)
27	9.81	C30H26O14			611.13867	-1.41	(Epi)gallocatechin-(epi)gallo-catechin	MS2[611@30]:127(83),139(100),179(37),287(48),425(5)
28	10.10				259.11459		Unidentified	MS2[259@30]:161(5),227(3)
29	10.15		405.04822				Unidentified	MS2[405@30]:97(18),119(53),163(100),241(14)
30	10.16	C30H26O14	609.12299	-1.464	611.13892	-1.001	(Epi)gallocatechin-(epi)gallo-catechin	MS2[611@30]:127(4),139(100),163(89),179(3),287(6),425(5)
31	10.20	C37H30O18	761.13318	-2.181			(Epi)gallocatechin-(epi)gallo-catechingallate	MS2[761@30]:125(100),137(17),161(10),169(14),177(39),305(6)
32	10.52				259.11469		Unidentified	MS2[259@30]:85(7),227(3)
33	10.79				407.09415		Unidentified	MS2[407@30]:85(8),107(8),139(25),188(100),205(19),233(3)
34	10.80	C45H38O21	913.18127	-1.001	915.19763	-0.223	(Epi)gallocatechin-(epi)gallo-catechin-(epi)gallocatechin	MS2[913@30]:125(100),137(14),167(10),177(50),243(13) MS2[915@30]:139(28),153(74),245(18),287(100),425(5)
35	10.88	C11H12O2N2			205.09671	-2.166	Tryptophan	MS2[205@30]:146(54),147(8),188(100)

Table S1. *Cont*.

36	11.03	C11H20O5N2	259.12885	0.007	261.14377	-2.789	Glutamyl-leucine(Glu-Leu)	MS2[259@30]:62(5),103(6),128(100),130(68),147(11)，179(3),197(5),223(4),241(2) MS2[261@30]:84(24),86(100),130(16),132(60),198(20)，244(10)
37	11.34	C20H36O11	451.21686	-1.171			Glucosyloctenylglucose	MS2[451@30]:59(38),71(43),89(100),101(45),119(32),192(8),216(8).394(8)
38	11.41	C12H14O8	285.06036	-0.469			Dihydroxybenzoic acid xyloside	MS2[285@30]:108(96),109(18),152(100),153(82)
39	11.46				429.20868		Unidentified	MS2[429@30]:99(3)
40	11.67				259.11459		Unidentified	MS2[259@30]:86(100),130(66),198(16),244(13)
41	11.72	C11H20O5N2	259.12866	-0.726	261.14349	-3.861	Glutamylleucine(Glu-Leu)	MS2[259@30]:128(100),130(61),197(6),223(5),241(5) MS2[261@30]:84(26),86(100),130(17),132(61),198(16)，244(12)
42	11.88	C7H6O4	153.01730	-6.111			Dihydroxybenzoic acid	MS2[153@30]:85(15),95(13),108(36),109(100),123(11)
43	12.05		405.04803				Unidentified	MS2[405@30]:97(16),119(53),163(100),241(15)
44	12.32	C37H30O18	761.13318	-2.181	763.1496	-1.167	(Epi)gallocatechin-(epi)gallo-catechingallate	MS2[761@30]:125(100),137(9),161(10),169(11),177(36),243(5),305(5)
45*	12.37	C7H6O3	137.02245	-6.353			4-Hydroxybenzoic acid	MS2[137@30]:93(100)

Table S1. *Cont*.

46	12.44				209.15317		Unidentified	MS2[209@30]:83(69),151(74),167(100)
47	12.44				267.15585		Unidentified	MS2[267@30]:239(6),241(12),259(5)
48	12.69		413.16501				Unidentified	MS2[413@30]:71(68),101(87),136(39),161(46),235(100)
49	12.75	C7H6O4	153.01746	-5.066			Dihydroxybenzoic acid	MS2[153@30]:85(6),95(5),108(11),109(100),135(18)
50	12.79				391.15674		Unidentified	MS2[391@30]:71(10),86(5),149(46),167(3)
51	12.83	C8H8O5	183.02815	-3.55			Methylgallic acid	MS2[183@30]:71(10),86(5),149(46),167(3)
52	13.02	C23H22O12	489.10223	-1.068			Pectolinaringenin glucuronoside	MS2[489@30]:62(5),113(9),125(18),163(8),183(100),305(3)
53	13.08	C15H14O7	305.06549	-0.292	307.08041	-2.668	(Epi)gallocatechin	MS2[305@30]:109(9),125(100),137(19),179(2),167(15)
54	13.30	C36H32O19	767.14398	-1.857			Myricitrinyl-(epi)gallo-catechin	MS2[767@30]:125(100),137(10),169(15),177(39),243(10),316(8),527(3)
55	13.42				229.10657		Unidentified	MS2[229@30]:83(6),101(3),111(4),129(3),167(100),181(5),193(3)
56	13.55		284.02228				Unidentified	MS2[284@30]:80(10),97(21),142(63),158(10),186(6),204(100),205(16),210(14),222(22)

Table S1. *Cont*.

57	13.57	C21H32O10	443.19055	-1.407			Plucheoside C or its isomers	MS2[284@30]:80(10),97(21),142(63),158(10),186(6),204(100),205(16),210(14),222(23)
58	13.69				429.20895		Unidentified	MS2[429@30]:99(15),203(20)
59	13.78				467.18823		Unidentified	MS2[467@30]:139(5),203(2),287(6),305(2),347(4),449(8)
60	13.83	C12H22O8	293.12311	0.054			Stophanthobiose or its isomers	MS2[293@30]:59(19),71(25),89(36),101(28),113(12),119(15),131(100)
61	13.92	C15H18O8	325.09158	-0.658			Coumaric acid glucoside	MS2[325@30]:85(6),119(70),`47(5),163(100),191(3)
62	14.35	C12H21O8	293.12323	0.464			Stophanthobiose or its isomers	MS2[293@30]:59(19),71(25),89(39),101(28),113(13),119(18),131(100)
63	14.65	C44H34O22	913.14435	-1.587	915.16028	-1.277	(Epi)gallocatechingallate-(epi) gallocatechin gallate	MS2[913@30]:125(100),137(6),161(11),167(16),177(53),243(5),285(8),305(3) MS2[915@30]:139(26),153(72),179(8),245(17),287(100),299(29),425(5)
64	14.84	C19H28O12	447.14896	-1.661			O-Acetylshanzhiside methyl ester or its isomers	MS2[447@30]:59(22),71(80),89(32),101(98),113(55),131(51),161(100),269(23)
65	14.97		399.00098				Unidentified	MS2[399@30]:125(42),151(6),153(4),175(23),179(20),193(100),215(5),301(5)

Table S1. *Cont*.

66	15.13				361.14612		Unidentified	MS2[361@30]:92(3),229(13)
67	15.13				425.14111		Unidentified	MS2[425@30]:92(2),293(10),317(3)
68*	15.35	C9H8O4	179.03316	-4.051			Caffeic acid	MS2[179@30]:59(24),71(45),89(30),113(25),135(100)
69	15.37	C20H36O11	451.21683	-1.237			Glucosyloctenylglucose	MS2[451@30]:59(38),71(63),89(100),101(58),113(36),119(42)
70	15.80	C19H28O12	447.14896	-1.661			O-Acetylshanzhiside methyl ester or its isomers	MS2[447@30]:59(20),71(100),89(42),101(96),113(46),161(89),269 (29)
71	16.05				425.1409		Unidentified	MS2[425@30]:92(2),293(10),317(3)
72	16.07	C42H38O24	925.16467	-2.441	927.18079	-1.929	Myricitrinyl-myricitrin	MS2[925@30]:151(53),179(100),193(6)<br>MS2[927@30]:85(49),153(53),301(9),319(8),607(5),635(100)
73	16.19	C20H32O10	431.19016	-2.351			Propylshanzhiside methyl ester or its isomers	MS2[431@30]:59(30),71(92),89(62),101(53),113(52),153(100),161 (15),205(15)
74	16.26	C22H18O11	457.07562	-2.008			(Epi)gallocatechin gallate	MS2[457@30]:125(45),169(100),179(2)
75	16.32	C18H32O12	439.17987	-2.579			Glucosylhexanoylglucose	MS2[439@30]:71(38),101(50),131(100),161(38),179(20),261(45)
76	16.42	C16H28O10N2			409.1821	1.047	N-(1-Deoxyfructopyranos-1-yl) -isoleucylaspartic acid or its isomers	MS2[409@30]:203(2)

Table S1. *Cont*.

77	16.65		261.00641				Unidentified	MS2[261@30]:163(23),191(8),205(8)
78*	16.69	C15H14O6	289.07062	-0.154	291.08566	-2.249	L-epicatechin	MS2[289@30]:81(16),97(25),109(85),125(74),137(49),151(24),179(15),203(25),223(39),247(100) MS2[291@30]:113(43),139(100),147(15),165(14),243(8)
79	16.77	C36H32O19	767.14398	-1.857			Myricitrinyl-(epi)gallocatechin	MS2[767@30]:113(33),125(70),151(52),169(20),179(100),273(5)
80	16.78	C11H18O2N6			267.15613	-1.012		MS2[267@30]:223(15),235(10),239(15),241(22),253(10)
81	16.88	C20H32O10	431.19049	-1.585			Propylshanzhiside methyl ester or its isomers	MS2[431@30]:59(40),71(62),89(100),101(53),113(52),153(15),161(5),179(8)
82	16.95		399.00122				Unidentified	MS2[399@30]:125(42),151(6),153(5),175(23),179(20),193(100),215(5),301(5)18
83	17.05	C16H28O10N2			409.18228	1.487	N-(1-Deoxyfructopyranos-1-yl) isoleucylaspartic acid or its isomers	MS2[409@30]:203(55),229(32)
84	17.30	C18H32O12	439.18048	-1.19			Glucosylhexanoylglucose	MS2[439@30]:71(38),101(50),131(100),161(38),179(20),261(45)
85	17.52	C10H17O4N	214.10701	-1.749			Monascumic acid or its isomers	MS2[214@30]:130(100),131(8),142(8),152(3),170(3)

Table S1. *Cont*.

86	18.07		269.13837				Unidentified	MS2[269@30]:177(100)
87	18.19				449.28891		Unidentified	MS2[449@30]:85(24),95(100),113(28),149(48),161(24),189(25),207(54)
88	18.30	C8H14O5	189.07513	-3.279			Butoxysuccinic acid or its isomer	MS2[189@30]:85(100),86(6),87(1),115(15)
89	18.31	C11H14O7	257.06320	-2.379			Chinensisol or its isomers	MS2[257@30]:85(100),86(6),113(30),115(32)
90	18.40	C22H18O11	457.07620	-0.739			(Epi)gallocatechin gallate	MS2[457@30]:125(45),169(100),179(2)
91	18.52	C10H17O4N	214.10707	-1.469			Monascumic acid or its isomers	MS2[214@30]:99(5),130(100),131(8),142(8),152(3),170(3)
92	18.54		217.10669				Unidentified	MS2[217@30]:59(8),83(15),127(15),137(50),155(100),173(25),181(23),217(58)
93	19.03	C36H28O20	779.10736	-2.13			Myricetinylmyricitrin	MS2[779@30]:125(20),151(79),169(16),179(100),193(8)
94	19.08	C9H8O3	163.03812	-0.851	165.05438	-1.458	Coumaric acid	MS2[163@30]:119(100)
95	19.17	C36H32O19	767.14374	-2.17			Myricitrinyl-(epi)gallocatechin	MS2[767@30]:125(43),151(49),163(13),167(20),179(100),273(6)
96	19.19		138.01768				Unidentified	MS2[138@30]:108(26)
97	19.21	C18H32O12	439.18030	-1.6			Glucosylhexanoylglucose	MS2[439@30]:71(38),101(50),131(100),161(38),179(20),261(45)
98	19.22	C49H42O28	1077.17542	-2.29			Myricitrinyl-myritrin gallate	MS2[1077@30]:125(30),151(38),169(40),179(20),193(14)

Table S1. *Cont*.

99	19.33				307.11456		Unidentified	
100	19.59	C8H14O5	189.07518	-3.015			Butoxysuccinic acid or its isomer	MS2[189@30]:85(100),86(6),87(1),115(15)
101	19.65	C20H32O10	431.19061	-1.307			Propylshanzhiside methyl ester or its isomers	MS2[431@30]:59(44),71(63),89(100),101(52),113(36),119(35),161(5),179(4)
102	19.77	C16H28O10N2			409.18253	2.098	N-(1-Deoxyfructopyranos-1-yl) isoleucylaspartic acid or its isomers	MS2[409@30]:203(26),213(10)
103	19.79	C12H8O6	247.02356	-0.625			2-Acetyl-3,5,8-trihydroxy-1,4-naphthoquinone	MS2[247@30]:113(73)
104	19.98	C41H38O23	897.17059	-1.587			Myricitrinylquercetin-arabinoside	MS2[897@30]:125(9),151(35),179(100),193(6),762(6),853(5)
105	19.99	C11H16O3			197.11684	-1.932	Methoxyhydroxythymol	MS2[197@30]:107(25),135(55),179(95)
106	20.12	C18H32O12	439.18048	-1.19			Glucosylhexanoylglucose	MS2[439@30]:71(38),101(50),131(100),161(38),179(20),261(45)
107	20.38	C36H32O19	767.14392	-1.936			Myricitrinyl-(epi)gallocatechin	MS2[767@30]:125(15),151(53),169(14),179(100),273(3)
108	20.52	C36H28O20	779.10767	-1.732			Myricetinylmyricitrin	MS2[779@30]:125(20),151(80),169(18),179(100),193(6)
109	20.52	C41H38O23	897.17053	-1.654			Myricitrinyl-quercetin-arabinoside	MS2[897@30]:125(8),151(32),179(100),193(6),393(9)
110	20.75	C10H10O4	193.04893	-3.135			Ferulic acid	MS2[193@30]:134(100),149(15),178(25)

Table S1. *Cont*.

111	20.96	C49H42O28	1077.17590	-1.844			Myricitrinylmyritrin gallate	MS2[1077@30]:125(13),151(48),169(18),179(100),193(6)
112	21.54	C10H16O7	247.08112	-0.442			2-Methyl-3-[2,3,5-trihydroxy-6-(hydroxymethyl)tetrahydro-2 H-pyran-2-yl]-2-propenoic acid	MS2[247@30]:87(39),111(100),113(33),185(6)
113	21.54	C20H24O8	391.13852	-0.573			Hamigeromycin B or its isomer	MS2[391@30]:135(12),150(48),165(100),180(3),195(70)
114	22.06	C41H36O23	895.15479	-1.758			Quercitrinylquercetin-galactoside	MS2[895@30]:125(13),151(13),179(13),253(3),301(100),486(3),628(3)
115	22.12	C10H18O5N2			247.12993	4.378	Glutamyl-valine (Glu-Val)	MS2[247@30]:189(6),211(2)
116	22.22	C20H24O8	391.13831	-1.11			Hamigeromycin B or its isomer	MS2[391@30]:135(2),150(23),165(100),180(6),195(46)
117	22.47	C7H6O3	137.02240	-6.718			Hydroxybenzoic acid	MS2[137@30]:93(100)
118	23.35	C25H40O13	547.23737	-2.097			Megastigma-diglucoside	MS2[547@30]:59(33),71(47),89(100),101(32),131(32),149(23),191(18)
119	23.54	C15H12O6	287.05478	-0.817			Dimethoxy-dihydroxy-xanthone	MS2[287@30]:125(100),151(9),223(53),243(5),259(13)
120*	23.66	C21H20O12	463.08621	-1.927	465.10141	-1.342	Myricitrin	MS2[463@30]:151(20),179(54),235(12),316(100),377(10) MS2[465@30]:71(45),85(48),319(100)
121	23.66		387.06375				Unidentified	MS2[387@30]:80(23),116(54),120(72),134(26),178(100),237(26),263(16),307(27)

Table S1. *Cont*.

122	23.69	C26H36O11			525.22955	-6.641	Secoisolariciresinol glucoside	MS2[525@30]:333(6),335(10),393(3)
123	24.06	C49H42O28	1077.17590	-3.543			Myricitrinylmyritrin gallate	MS2[1077@30]:151(83),169(8),179(18),285(50),316(100),461(75)
124	24.23	C26H36O11			525.22931	-7.098	Secoisolariciresinol glucoside	MS2[525@30]:333(5),335(6)
125	24.25				445.14612		Unidentified	MS2[445@30]:189(5),248(100),359(7)
126	24.46	C25H22O2			355.17169	6.852	Maculalactone A	MS2[355@30]:203(5)
127	24.59	C20H32O9	415.19589	-0.889			Cassioside or its isomers	MS2[415@30]:71(52),89(100),101(38),113(25),283(4),297(3)
128	24.73	C19H30O7			371.20563	-2.154	Byzantionoside A or its isomer	MS2[371@30]:109(14),133(100),137(63),149(13),191(25)
129	24.79	C19H24O8N2			409.16147	2.268	Urauchimycin C	MS2[409@30]:59(4),77(5),85(5),149(3),165(3),169(3)
130	24.97				527.24567		Unidentified	MS2[527@30]:355(4)
131	25.26		300.99777				Unidentified	MS2[301@30]:88(9),102(8),180(9),210(7)
132	25.40	C9H16O4	187.09589	-3.183			Eucommiol	MS2[187@30]:85(5),97(8),125(100),169(2)
133	26.16	C14H24O6	287.14893	0.053			Undecanetricarboxylic acid	MS2[287@30]:59(100),223(5),227(7)

Table S1. *Cont.*

134	26.34	C28H24O16	615.09674	-2.147	617.1131	-0.99	Myricitrin gallate	MS2[615@30]:125(8),137(11),151(33),169(100),179(63),289(5),317(55); MS2[617@30]:153(100),319(15)
135	26.38				311.14572		Unidentified	MS2[311@30]:249(11),293(5)
136	26.81	C44H38O22	917.17169	-5.897	919.18896	-3.85	Quercitrinyl-(epi)gallocatechin methyl gallate	MS2[917@30]:125(8),151(18),179(23),301(100) MS2[919@30]:169(27),325(100),471(20)
137	26.83	C15H18O3			247.13225	-2.513	Lacinilene C or its isomers	MS2[247@30]:173(63),187(100),201(70),219(15),229(43)
138	26.84				287.12463		Unidentified	MS2[287@30]:121(6),217(8)
139	26.88	C21H20O11	447.09131	-1.963	449.10699	-1.888	Quercitrin	MS2[447@30]:151(55),179(49),300(100),301(75),361(8) MS2[449@30]:71(36),85(39),129(8),303(100)
140	27.05	C28H24O16	615.09650	-2.538	617.11261	0.392	Myricitringallate	MS2[615@30]:125(7),137(25),151(45),169(53),179(75),283(5),317(100) MS2[617@30]:153(100),319(5)
141	27.24	C13H20O2			209.15315	-2.182	3-Hydroxy-β-ionone or its isomers	MS2[209@30]:109(17),149(15),165(100),191(13)
142	28.04	C11H13O5	225.07535	-1.777			Trimethoxybenzoic acid methyl ester	MS2[225@30]:71(11),89(18),125(20),169(33)
143	28.12	C28H30O10			527.18799	-6.039	Punarnavoside	MS2[527@30]:365(5)

Table S1. *Cont*.

144	28.14	C13H18O			191.14258	-2.416	Damascenone or its isomers	MS2[191@30]:121(8),133(100),137(28),149(12)
145	28.15	C13H20O2			209.15315	-2.182	3-Hydroxy-β-ionone or its isomers	MS2[209@30]:109(17),133(97),137(100),149(18),165(5),173(10),191(13)
146	28.97	C23H22O12	489.10226	-1.007	491.11783	-1.166	Acetylquercitrin	MS2[489@30]:89(30),113(99),125(15),151(28),179(25),300(100)<br>MS2[491@30]:71(53),111(76),129(100),303(60),491(20)
147	29.00	C22H38O10	461.23779	-0.724			Geranyl rutinoside or its isomers	MS2[461@30]:71(80),89(100),101(60),113(43),161(10),314(15)
148	29.21	C21H20O10	431.09653	-1.724			Kaempferol rhamnoside	MS2[431@30]:59(32),113(25),149(8),165(36),221(6),285(100)
149	29.49	C14H20O5	267.12286	0.598			Pentylurofuransaeure	MS2[267@30]:59(100),71(4),207(2),223(3)
150	29.72	C23H22O12	489.10217	-1.191	491.11765	-1.532	Acetylquercitrin	MS2[489@30]:71(40),89(100),113(99),125(8),151(23),179(15),300(66)<br>MS2[491@30]:69(66),111(36),129(66),303(100),491(10)
151	29.72		507.20621				Unidentified	MS2[507@30]:71(48),89(100),101(23),113(9),125(20),167(53),191(5)
152	30.15				485.19852		Unidentified	MS2[485@30]:191(3),229(5),317(100)

Table S1. *Cont*.

153*	30.58	C20H22O5			343.15323	-2.245	(2R)-3',4''-epoxy-2-hydroxy-1-(4-hydroxyphenyl)-7-(3-methoxyphenyl)heptan-3-one	MS2[343@30]:107(70),123(83),131(100),161(56),191(53)
154	30.95		407.11530				Unidentified	MS2[407@30]:97(100),309(5)
155	31.09	C24H22O8	437.12561	5.756	439.14136	5.684	Kotanin or its isomers	MS2[439@30]:211(13),227(30),239(45),271(100),309(54),341(65)
156	31.09		897.24121				Unidentified	MS2[897@30]:97(100),111(6)
157	31.67	C18H30O5	325.20068	-0.832			Manadic acid B or its isomers	MS2[325@30]:85(63),97(32),171(100),211(40),229(20),291(5)
158	32.10	C18H32O5	327.21631	-0.888			Fulgidic acid or its isomers	MS2[327@30]:85(88),97(36),137(28),171(100),211(39),229(22),291(3)
159	32.34	C15H10O2N			237.0786	0.717	Unidentified	MS2[237@30]:223(18)
160	32.38	C25H34O8	461.21649	-1.094			Acetylstrophadogenin	MS2[461@30]:61(26),113(100),124(32),165(25),168(30),223(52),245(55)
161	32.45		297.04584				Unidentified	MS2[297@30]:75(12),149(100)
162	32.55		239.05843				Unidentified	MS2[239@30]:223(100)
163	32.55	C15H11O5N	285.06400	2.898			Unidentified	MS2[285@30]:223(100)
164	32.71	C11H20O13	359.08258	1.568			Unidentified	MS2[359@30]:74(8),91(100)
165*	32.80	C21H26O5			359.18423	-2.98	Myricanol	MS2[359@30]:211(14),227(13),259(27),271(100),309(30),341(11)
166	32.93				274.27322		Unidentified	MS2[274@30]:256(5)
167	32.98		461.21652				Unidentified	MS2[461@30]:113(100),223(52),245(56)

Table S1. *Cont*.

168	32.99	C11H14O7N4	313.07739	2.722		7-oxo-N(8)-(D-ribityl)-7,8-dihydrolumazin or it isomers	MS2[313@30]:74(12),91(100)
169	34.71	C11H14O7N4	313.07730	-1.837		7-oxo-N(8)-(D-ribityl)-7,8-dihydrolumazin or it isomers	MS2[313@30]:74(13),91(100)
170	35.71		433.10092			Unidentified	MS2[433@30]:91(5),149(23),165(100)
171	35.84				411.09274	Unidentified	MS2[411@30]:170(5),194(23),346(5),367(5)
172	35.91		165.03900			Unidentified	MS2[165@30]:75(14),91(5),149(100),150(10),151(5)
173	36.17				279.15833	Unidentified	MS2[279@30]:149(100)
174	36.79				371.10034	Unidentified	MS2[371@30]:73(100),91(74),285(70)
175	36.81		507.11948			Unidentified	MS2[507@30]:75(6),91(15),149(28),165(100),223(59)
176	36.81		223.02686			Unidentified	MS2[223@30]:89(100)
177	36.81		165.03899			Unidentified	MS2[165@30]:75(13),91(5),149(100),150(10),151(5)
178	36.95		297.04584			Unidentified	MS2[297@30]:75(10),149(100)
179	37.24	C11H14O7N4	313.07730	-1.741		7-oxo-N(8)-(D-ribityl)-7,8-dihydrolumazin or it isomers	MS2[313@30]:74(13),91(100)
180	37.31		581.13831			Unidentified	MS2[581@30]:75(8),91(101),165(16),223(25)
181	38.16	C11H14O7N4	313.07733	-1.837		7-oxo-N(8)-(D-ribityl)-7,8-dihydrolumazin or it isomers	MS2[313@30]:74(13),91(100)
182	38.22		655.15680			Unidentified	MS2[655@30]:75(8),91(100),149(5),165(24),223(5)
183	38.76		729.17535			Unidentified	MS2[729@30]:75(8),91(95),149(20),165(100),223(13)

Table S1. *Cont*.

184	38.81				707.16736	Unidentified	
185	39.18		803.19385			Unidentified	MS2[803@30]:75(8),91(93),149(20),165(100),223(43)
186	39.50		877.21265			Unidentified	MS2[877@30]:75(5),91(100),113(8),149(10),165(35),223(28)
187	39.50		951.23090			Unidentified	MS2[951@30]:75(5),91(100),113(15),149(15),165(63),223(26)
188	40.73	$C_{29}H_{48}O_8$	523.32910	4.883		Epigynoside C	MS2[523@30]:113(12),223(100)
